# May-Thurner Syndrome—a Rare Cause of Extensive Pelvic DVT, but Is there More to Know?

**DOI:** 10.1155/2022/7978470

**Published:** 2022-10-25

**Authors:** Vikash Kumar, Michelle Koifman, Bhavyakumar Vachhani, Dhir Gala, Sumeet Bahl

**Affiliations:** ^1^The Brooklyn Hospital Center, 121 DeKalb Ave, Brooklyn, NY 11201, USA; ^2^American University of the Caribbean School of Medicine, 1 University Drive at Jordan Dr, Cupecoy, Saint Martin

## Abstract

May-Thurner Syndrome (MTS) is a rare anatomical variant characterized by the compression of the left common iliac artery by the right common iliac artery against the fifth lumbar vertebrae. It can present as acute or chronic deep vein thrombosis (DVT), leg pain, varicosities, skin ulceration, and hyperpigmentation. In this case report, we present an interesting case of a young male with no obvious risk factors, who presented with back and left lower extremity pain later diagnosed with MTS on computed tomography angiography (CTA) and venogram. The patient was treated with venoplasty and pharmacomechanical thrombolysis and was discharged on apixaban.

## 1. Introduction

**Figure 1:**
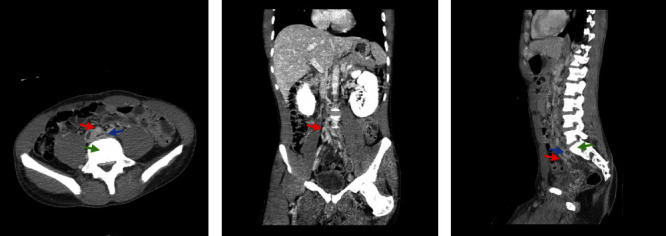
(a) CT abdomen/pelvis with contrast (axial view) showing obstructed vein (blue arrow) in between artery (red arrow) and vertebrae (green arrow). (b) CT abdomen/pelvis with contrast (coronal view) showing site of obstruction at left common iliac vein crossing in between right common iliac artery and L5 vertebrae. (c) CT abdomen/pelvis with contrast (lateral view) showing obstructed vein (blue arrow) in between artery (red arrow) and vertebrae (green arrow).

May-Thurner syndrome (MTS), coined after Dr. May R. and Dr. Thurner J., is a condition describing the phenomenon of sinistral occurrence of thrombosis of the pelvic veins due to extrinsic venous outflow obstruction. It has been defined as the compression of the left iliofemoral vein by the right common iliac artery against the vertebral body (Figure 1) [1]. It is a rare cause of extensive DVT, compared to the usual presentation of DVTs that are more commonly seen as a result of various underlying illnesses.

## 2. Case Presentation

**Figure 2:**
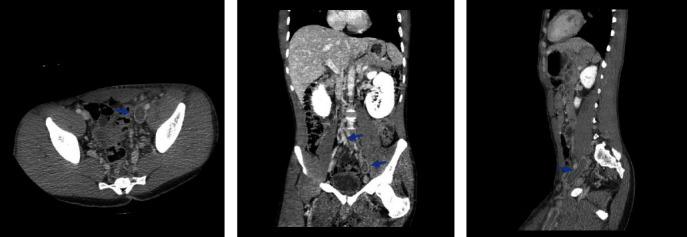
(a, b, c) CT abdomen/pelvis with contrast significant for occlusive thrombus in visualized portions of deep femoral veins, extending into the left common femoral vein, and into the left external, internal, and common iliac veins.

**Figure 3:**
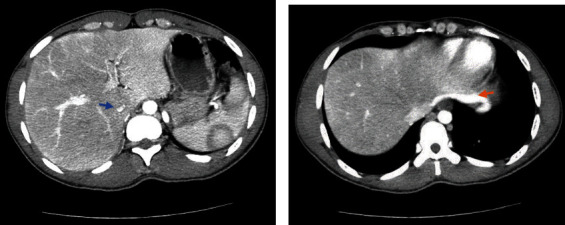
(a) CT abdomen/pelvis with contrast showing intrahepatic IVC stenosis (blue arrow). (b) Seen is a large diaphragmatic venous collateral to the IVC (orange arrow), indicating that the thrombosis in this patient was acute on chronic pathology.

**Figure 4:**
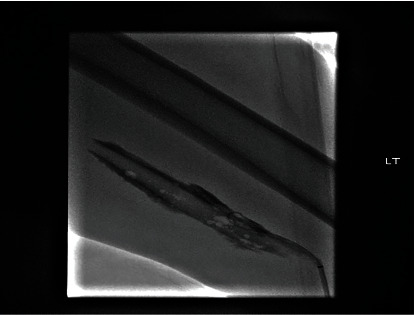
Venogram seen above displaying a “tram-track pattern”, which is significant for DVT in the stenosed vein.

A 28-year-old African American male with a past medical history of tobacco use disorder presented to the hospital with a 3-day history of lumbar back and posterior left lower extremity pain. He denied any recent trauma, prolonged immobilization, history of thromboembolic disease, or family history of thromboembolic disorders. Vitals were significant for a heart rate of 104 bpm. Physical examination revealed warmth and tenderness to palpation of the left thigh, with associated moderate nonpitting edema of the left lower extremity. Labs were significant for WBC of 10.9, platelet count of 743,000, CRP of 286.13, lactic acid of 2.6, and D-dimer that was >4.40. EKG showed LV hypertrophy and sinus tachycardia. Initial assessment with a point of care ultrasound to the left lower extremity showed noncompressible left common femoral vein, left saphenofemoral junction, left superficial and deep femoral veins confluence, and left popliteal vein. CTA chest was unremarkable for pulmonary embolism or any other pulmonary pathology. CTA abdomen/pelvis showed occlusive thrombus in visualized portions of left superficial and deep femoral veins, extending into the left common femoral vein, and into the left external, internal, and common iliac veins, with compression of the left common iliac vein by the crossing right common iliac artery, findings were suggestive of MTS (Figure 2). There was also left para-aortic and left external iliac lymphadenopathy, possibly reactive, however metastatic disease could not be excluded. CTA abdomen/pelvis also showed an unusually narrow inferior vena cava (IVC) indicative of intrahepatic IVC stenosis (Figure 3(a)), with multiple intra-abdominal collaterals (Figure 3(b)). The Hematology-Oncology team was consulted in concern of extensive lymphadenopathy and thrombocytosis, who further recommended workup, including PET-CT, to rule out malignancy. JAK2 mutation, CALR, and MPL testing ruled out a myeloproliferative neoplasm. Workup for thrombophilia (antiphospholipid antibodies, anticardiolipin antibodies, anti-beta-2-glycoproteins, Protein C and S, and homocysteine) was negative. The patient was started on therapeutic enoxaparin and switched to a heparin drip as per the Interventional Radiology (IR) team's recommendations. The patient underwent a left lower extremity venogram by the IR team which showed an occluded left popliteal vein extending up to the left external iliac vein with a tram-track pattern (Figure 4). The patient was then transferred to a specialty facility where he underwent a repeat venogram with venoplasty and pharmacomechanical thrombectomy. He was further moved to the Medical ICU for close monitoring postoperatively. He was discharged on postoperative day 3 on apixaban for at least 6 months with close outpatient follow-up to determine the duration of anticoagulation.

## 3. Discussion

DVT is a common and potentially life-threatening problem, with the incidence of it varying with age. Annual incidence rates by age group is approximately 2–3 per 10,000 (30–49 years), 5 per 10,000 (50–59 years), 10 per 10,000 (60–69 years), and 20 per 10,000 (70–79 years) [2]. DVT usually occurs in the setting of predisposing factors such as recent surgery or trauma, immobilization of limbs, active cancer, acute medical illnesses, obesity, use of oral contraceptive medications, and thrombophilic disorders [3].

Venous stasis has been long known to be a predisposing factor for thrombosis and is part of the famous Virchow triad along with hypercoagulability and endothelial injury. One cause of venous stasis was first described in 1957 by May and Thurner during their study of 430 cadavers, where they found spur-like projections within the venous wall of 15–22% of the investigated cases. These spurs, which were believed to be a result of irritation induced by the pulsation of the overlying artery, cause luminal narrowing and promote clot formation leading to thrombosis.

Based on a systematic review, the mean age of presentation for MTS is 25–50 years, more common in females with a female to male ratio of 2–4.7 : 1 [4], and most commonly involving the left side as the left common iliac vein crosses between the right common iliac artery and the L5 spine [5]. MTS presentation can vary from mild swelling or severe extensive DVT and PE. MTS is often overlooked in young patients without risk factors due to several reasons. Firstly, by review of the literature, there are very few cases of male patients presenting with DVT due to May-Thurner syndrome. Furthermore, MTS may be missed as a potential alternative diagnosis when common risk factors of DVT, such as prolonged bed rest, postsurgery, malignancy, or oral contraceptive pill use [6] are already confirmed and further workup is not performed. Finally, the low incidence of diagnosis is also related to the fact that a usual DVT is commonly diagnosed with an ultrasound, whereas diagnosing the defect that develops with MTS, which usually occurs higher in the pelvis, requires more specific studies such as a CT scan or an MRI [7].

Early diagnosis and prompt treatment of DVT due to MTS is essential. Diagnosis modalities include plain CT, CTA, however, IVUS with conventional venography is the gold-standard modality to diagnose MTS [8]. This can provide accurate sizing of the luminal diameter and even provide insights into the chronicity of the thrombus with the presence or absence of collateral venous and fibrotic changes. In our case, CTA abdomen/pelvis and venogram findings were consistent with the diagnosis of MTS. In addition, intrahepatic IVC stenosis with multiple collaterals was also seen. While the exact cause of the oddly narrow IVC is not obvious, a possible differential could include a congenital anomaly or consequences of chronic thrombosed lower extremity veins in our patient. What was initially thought to be MTS alone, was actually understood to be a stenotic IVC that in addition to the MTS itself led to thrombosis.

Treatment of MTS is guided by clinical presentation. It is generally accepted that a more invasive therapeutic approach is needed to prevent long-term-sequelae, as systemic anticoagulation may be insufficient [9]. Even though open surgery was used in the past, more recently, endovascular procedures such as catheter-directed thrombolysis and stent placement have been the preferred choice for treatment. Following stent placement, therapeutic anticoagulation is indicated, but there is no consensus regarding the optimal duration, type, and intensity of anticoagulation needed. For most patients, 6–12 months of anticoagulation following stent placement is usually sufficient.

Temporary IVC filters are yet another endovascular approach that may be considered in MTS. Similar to our young male patient, Hng et al. discussed the case of a 23-year-old male who presented with MTS. He underwent IVC filter placement, mechanical thrombectomy, thrombolysis, and further subcutaneous anticoagulation with the eventual removement of the IVC filter [10]. It is necessary to remove IVC filters as soon as possible to prevent filter-related DVT. As per a quantitative decision analysis, the benefit/risk profile is favorable for filter removal between 29 and 54 days after implantation [11].

## 4. Conclusion

While uncommon itself, MTS is even more rare in males. We believe the intrahepatic IVC stenosis in our young patient, together with the MTS lesion, led to a thrombophilic state triggering the thrombosis. The extensive collaterals identified on the CTA were indicative that the thrombosis was an acute event on a chronic pathology.

Considering the extensive nature of associated thrombosis, MTS if left untreated could lead to devastating consequences such as postthrombotic syndrome and chronic venous insufficiency. It is necessary to identify patients with high risk for MTS, especially younger individuals including males presenting with left-sided lower extremity swelling. Early recognition using US or CT venography techniques is necessary to prevent PE. Treatment involves anticoagulation, as well as endovenous management, including thrombolysis/thrombectomy, venous angioplasty, and stenting.

## Data Availability

No data were used to support this study.
